# A Lightweight Edge-Deployable Framework for Intelligent Rice Disease Monitoring Based on Pruning and Distillation

**DOI:** 10.3390/s26010035

**Published:** 2025-12-20

**Authors:** Wei Liu, Baoquan Duan, Zhipeng Fan, Ming Chen, Zeguo Qiu

**Affiliations:** 1 Harbin University of Commerce, Harbin 150028, China; weiwei@hrbcu.edu.cn (W.L.); duanbaoquan@hrbcu.edu.cn (B.D.); chenming@hrbcu.edu.cn (M.C.); qiuze@hrbcu.edu.cn (Z.Q.); 2 Heilongjiang Provincial Key Laboratory of Electronic Commerce and Information Processing, Harbin 150028, China

**Keywords:** digital agriculture, rice leaf disease detection, lightweight YOLO, network pruning, knowledge distillation

## Abstract

Digital agriculture and smart farming require crop health monitoring methods that balance detection accuracy with computational cost. Rice leaf diseases threaten yield, while field images often contain small multi-scale lesions, variable illumination and cluttered backgrounds. This paper investigates SCD-YOLOv11n, a lightweight detector designed with these constraints in mind. The model replaces the YOLOv11n backbone with a StarNet backbone and integrates a C3k2-Star module to enhance fine-grained, multi-scale feature extraction. A Detail-Strengthened Cross-scale Detection (DSCD) head is further introduced to improve localization of small lesions. On this architecture, we design a DepGraph-based mixed group-normalization pruning rule and apply channel-wise feature distillation to recover performance after pruning. Experiments on a public rice leaf disease dataset show that the compressed model requires 1.9 MB of storage, achieves 97.4% mAP@50 and 76.2% mAP@50:95, and attains a measured speed of 184 FPS under the tested settings. These results provide a quantitative reference for designing lightweight object detectors for rice disease monitoring in digital agriculture scenarios.

## 1. Introduction

Rice is a staple food for more than half of the world’s population, and its yield is directly linked to food security and social stability [1]. Major diseases such as bacterial leaf streak, rice blast and brown spot can cause substantial yield losses and even local crop failures [2,3]. Traditional field scouting and visual diagnosis are inefficient, highly subjective and strongly dependent on expert experience, making them inadequate for continuous monitoring across large rice-growing areas [4]. Unmanned aerial vehicle (UAV)-based hyperspectral remote sensing combined with machine learning algorithms and conventional methods such as support vector machines (SVM) has achieved promising results in crop growth assessment and yield prediction [5,6]. However, these approaches mainly focus on field-scale analysis and rely heavily on hand-crafted features, which makes them difficult to apply directly to fine-grained, leaf-level lesion detection and real-time early warning in rice fields.

Deep learning-based object detection has opened up new possibilities for intelligent crop disease identification [7,8]. In particular, the YOLO (You Only Look Once) family, as a representative single-stage detection framework, performs object localization and classification in a single forward pass and directly outputs bounding boxes and class probabilities on a dense grid. This design yields high accuracy and real-time speed, making YOLO-type detectors suitable for deployment on resource-constrained edge devices [9]. From YOLOv5 to YOLOv12, as well as newer variants such as YOLO-MS and Gold-YOLO, researchers have continually optimized the trade-off between detection accuracy and inference latency [10,11,12,13,14,15,16,17]. In the agricultural domain, a number of YOLO-based models have been proposed for rice and other crop disease detection, achieving improved performance through attention mechanisms, feature fusion structures and lightweight network designs [18,19,20,21,22]. Meanwhile, pruning and knowledge distillation techniques have been leveraged to compress YOLO-based detectors, significantly improving inference efficiency while preserving accuracy [22,23,24].

It should be noted that most existing disease detection methods are still evaluated primarily on desktop GPUs or under ideal laboratory conditions, with limited consideration of the computational, memory and energy constraints of edge devices deployed in real rice fields [18,19,20,21,22]. On the one hand, many lightweight YOLO variants are designed mainly for generic object detection and focus on reducing parameter counts and FLOPs, while lacking task-specific architectural designs for small, multi-scale and highly similar rice lesions in complex field environments [17,18,19,20]. On the other hand, pruning and channel-wise knowledge distillation (CWD) are typically adopted as generic compression tools; only a few studies integrate DepGraph-constrained structural pruning and channel-wise distillation within a unified scheduling framework explicitly aimed at optimizing the joint trade-off between accuracy and latency [22,23,24]. In addition, rice field images often exhibit strong illumination variations, interference from weeds and soil backgrounds, and co-occurrence of multiple lesion types. When only generic lightweight strategies are applied, it is difficult to simultaneously ensure high detection accuracy for small lesions and real-time inference on edge devices [4,5].

To address these issues, this study proposes an edge-deployable rice disease detection framework, SCD-YOLOv11n, for smart agriculture scenarios. Built on a StarNet backbone, the framework introduces a mixed group-normalization pruning rule and a DepGraph-driven structural pruning strategy, and combines them with channel-wise distillation (CWD) to construct a joint pruning–distillation schedule that achieves a better balance between accuracy and latency. A DSCD detection head is further designed to enhance fine-grained modeling of multi-scale lesion features, enabling customized optimization for complex paddy-field scenes. Unlike existing generic lightweight YOLO variants, the core of this work lies in designing a framework explicitly constrained by rice disease monitoring and in-field edge deployment, and in systematically exploring the synergy among structural pruning, channel-wise distillation and detection head design in this specific agricultural scenario. This provides methodological guidance for building reliable, low-latency, intelligent rice disease monitoring systems. The main contributions of this study are summarized as follows:

(1) We propose a mixed group-normalization pruning rule under the DepGraph framework that preserves channels sensitive to small lesions under a given compression ratio.
(2) We design a DSCD detection head with detail-enhancement and cross-scale branches to improve the detection of small and multi-scale rice lesions.
(3) We introduce a staged training strategy that combines structural pruning and channel-wise distillation (CWD) to jointly optimize accuracy, latency and model size.

## 2. Related Work

Vision-based rice disease monitoring has evolved from traditional field scouting toward smart farming systems that integrate sensing, automation and decision support [1,2,3]. Early methods relied on visual severity estimation together with hand-crafted color, texture and shape features, which were combined with classical classifiers such as SVM and k-nearest neighbors (k-NN) [4,5,6]. Recent surveys indicate that deep learning has become the mainstream approach for plant and rice disease recognition, covering both image-level classification and detection frameworks [3,7,8]. These approaches improve robustness compared with traditional methods; however, many existing models are still designed for image-level recognition or assume powerful GPUs, and their suitability for fine-grained lesion localization and real-time inference on resource-constrained edge devices in complex paddy fields remains limited.

Building on general-purpose detectors in the YOLO family [9,10,11,12,13,14,15,16,17], several task-specific models have been developed for crop and rice disease detection, such as YOLOv8-Rice, TLI-YOLO, SSD-YOLO and YOLO-CRD, which tailor anchors, feature fusion structures and lightweight modules to agricultural scenes [18,19,20,21]. YOLOv8-DDS further combines pruning and distillation for early disease detection in barley seedlings [22]. In parallel, structural pruning frameworks such as DepGraph and channel-wise knowledge distillation for dense prediction provide generic tools for compressing detectors [23,24]. Nevertheless, most pruning rules and distillation strategies are designed from a general perspective and are rarely co-optimized with detection head design for small, multi-scale rice lesions under strict edge-device constraints. These limitations directly motivate the task- and deployment-aware SCD-YOLOv11n framework proposed in this study.

## 3. Materials

**Figure 1:**
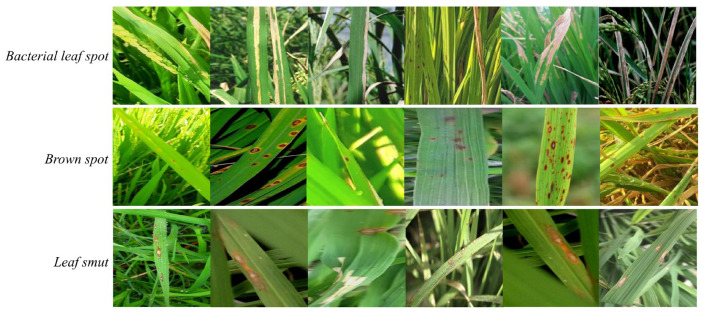
Representative image samples from the rice disease dataset.

In this study, all experiments are conducted on a public rice leaf disease image dataset containing three categories: bacterial leaf spot, brown spot and leaf smut. The dataset includes 6715 images in total, with 2863, 1643 and 2209 samples for each category, respectively. Images were collected in real paddy fields under varying illumination and background conditions, and were annotated by domain experts according to the YOLO bounding-box format. The dataset is randomly divided into training, validation and test sets with fixed proportions, and the same split is used for all models to ensure fair comparison. Representative samples from each class are shown in Figure 1, illustrating the small-scale, multi-scale and visually similar lesion patterns that make the task challenging. Additional information can be accessed to the supplementary file.

## 4. Methods

### 4.1. SCD-YOLOv11 Network Architecture

**Figure 2:**
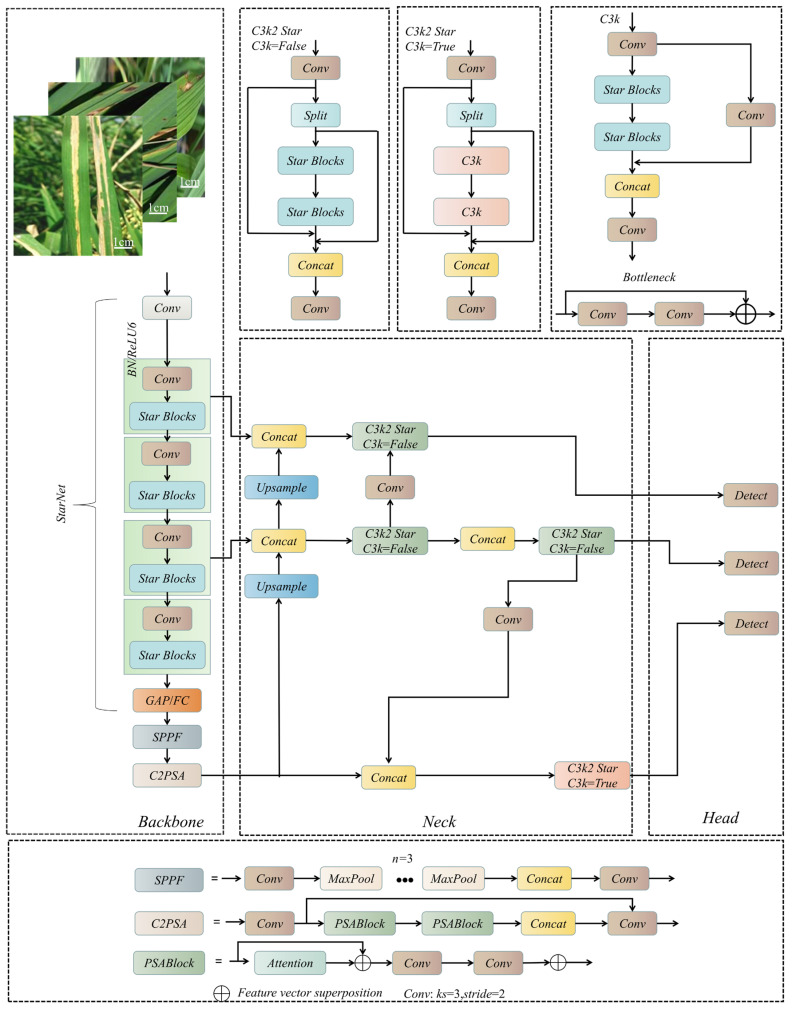
Structure of the SCD-YOLOv11 object detection network. The top-left rice leaf example includes a white scale bar, which approximately corresponds to 1 cm in real-world dimensions.

As illustrated in Figure 2, SCD-YOLOv11n follows the typical “backbone–neck–head” three-stage paradigm of the YOLO family and mainly consists of a StarNet backbone, a feature pyramid neck and a DSCD detection head. The overall design objective is to accurately detect rice leaf lesions of different sizes under complex field conditions, while satisfying the memory and latency constraints of edge devices.

The StarNet backbone replaces the original YOLOv11n backbone and is responsible for extracting fine-grained texture and edge features from rice leaves under varying illumination and cluttered backgrounds. The neck preserves the multi-scale feature fusion pathway, aggregating features at different resolutions in a top-down and bottom-up manner. On this basis, the DSCD head introduces dedicated detail-enhancement and cross-scale branches to improve the detection of small and multi-scale lesions. Built on this structural foundation, a DepGraph-based mixed group-normalization pruning scheme and channel-wise knowledge distillation (CWD) are further incorporated during training. These techniques reduce model parameters and improve inference efficiency on edge devices without changing the overall topology in Figure 2, thereby achieving a more balanced trade-off among detection accuracy, model size and inference speed.

### 4.2. StarNet Network Architecture and C3k2-Star

**Figure 3:**
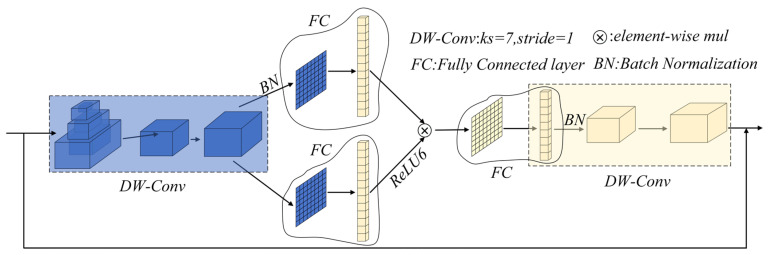
Architectural composition of the Star Block.

In paddy-field scenes, rice leaf lesions are typically small, fine-textured and low-contrast, and can be easily confused with veins, specular highlights and weeds in the background. To better capture such fine-grained patterns under the computational constraints of edge devices, we integrate the StarNet backbone [25] into the YOLOv11n framework and adapt it in a lightweight manner. StarNet is composed of a stack of Star Blocks, whose basic structure is illustrated in Figure 3. By introducing a learnable “star-shaped” operation on top of conventional convolutions, StarNet enhances the representational capacity of the backbone while maintaining a relatively low computational cost.

Let $x\in R^{d}$ denote the feature vector at a spatial location. The core star operation in a Star Block applies two linear projections to the same input and then performs an element-wise product between them:

(1) $$\phi \left(x\right)=\left(W_{1}^{T}x\right)⊙\left(W_{2}^{T}x\right)$$

where $W_{1},W_{2}\in R^{d\times d^{\prime }}$ are learnable projection matrices, and ⊙ denotes the Hadamard (element-wise) product. This operation implicitly models second-order interactions between channels, allowing each Star Block to encode richer nonlinear relationships with a parameter count comparable to that of a standard convolutional block, which is beneficial for distinguishing subtle differences between diseased and healthy leaf tissue.

At the network level, the StarNet backbone places Star Blocks at four spatial scales, with feature strides of 4, 8, 16 and 32. The corresponding channel widths are 16, 32, 64 and 128, and the numbers of Star Blocks at each scale are 1, 1, 3 and 1, respectively. Within a given scale, all Star Blocks share the same input and output channel widths, and only the internal 1 × 1 convolutions perform channel expansion and compression.

To integrate star-based feature extraction into the multi-scale neck while remaining compatible with YOLOv11n, we construct a C3k2-Star module by modifying the original C3 block. Let $F_{in}$ be the input feature map. The local branch and star branch are defined as:

(2) $${\;F}_{\mathrm{loc}}=\mathrm{Con}v_{3\times 3}\left(F_{\mathrm{in}}\right),\;F_{\mathrm{star}}=\mathrm{StarCon}v_{k}\left(F_{\mathrm{in}}\right)$$

where ${Conv}_{3\times 3}(\cdot )$ is a standard 3×3 convolution and ${StarConv}_{k}(\cdot )$ denotes a simplified Star Block whose effective receptive field is controlled by the kernel size k. The two branches are concatenated along the channel dimension and fused by a 1×1 convolution, followed by a residual addition with $F_{in}$:

(3) $$\;F_{out}\;=\;\mathrm{Con}v_{1\times 1}\left(\left[F_{loc},\;F_{star}\right]\right)+F_{in}$$

where $\left[\cdot ,\cdot \right]$ denotes channel-wise concatenation. This design introduces the star operation under a controlled number of parameters and FLOPs, improves the representation of small and slender rice lesions against complex leaf textures, and remains structurally compatible with the original C3 block so that it can be directly plugged into the YOLOv11n backbone.

### 4.3. Computational Architecture of the DSCD Lightweight Detection Head

**Figure 4:**
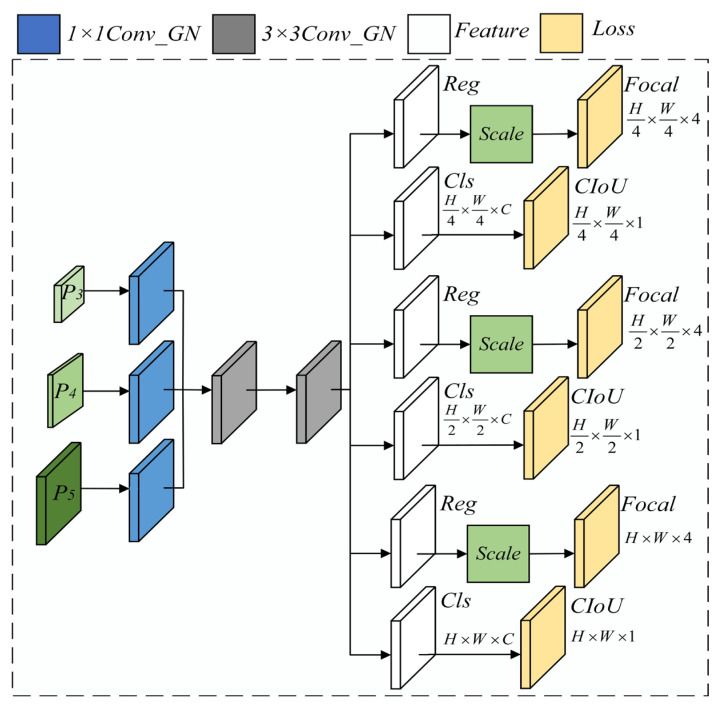
Architectural framework of the DSCD detection head.

In field images, rice leaf lesions are typically small, low-contrast and densely distributed, and different disease types often have very similar appearances. A standard YOLO detection head that mainly relies on coarse object boundaries tends to miss such lesions or confuse visually similar categories. To better adapt the detection stage to this scenario, we design a DSCD (Detail-Strengthened Cross-scale Detection) lightweight head on top of the YOLOv11n framework, as illustrated in Figure 4. The head preserves the three-scale prediction scheme, but enhances detail modeling through channel unification, a shared convolutional tower and a decoupled prediction structure.

The DSCD head takes as input three feature maps $\left\{P_{3},P_{4},P_{5}\right\}$ from the neck, corresponding to strides 8, 16 and 32 for small, medium and large lesions, respectively. For each scale, a 1×1 Conv_GN layer is first applied to compress and normalize the features to a unified channel width $C_{h}$:

(4) $$F_{l}^{\left(0\right)}={Conv}_{1\times 1}^{GN}\left(P_{l}\right),l\in 3,4,5$$

The three normalized feature maps are then passed through a shared stack of 3×3 Conv_GN layers to extract detection features:

(5) $$F_{l}=T\left(F_{l}^{\left(0\right)}\right)$$

where T(⋅) denotes a lightweight convolutional tower, whose parameters are shared across scales. Since the shared tower consists of two 3×3 convolutions with stride 1, the additional effective receptive field contributed by the DSCD head is approximately 5×5 on the feature maps. This corresponds to about 40×40, 80×80 and 160×160 pixels in the input image at strides 8, 16 and 32, respectively, which matches the typical size range of small, medium and large rice lesions.

Based on $F_{l}$, the DSCD head adopts a decoupled classification (Cls) and regression (Reg) structure, which can be abstractly written as

(6) $$S_{l}=f_{cls}\left(F_{l}\right),B_{l}=f_{reg}\left(F_{l}\right)$$

where $S_{l}\in R^{H_{l}\times W_{l}\times C}$ are the class logits for C disease categories, and $B_{l}\in R^{H_{l}\times W_{l}\times 4}$ are the bounding-box outputs. In implementation, $f_{cls}(\cdot )$ and $f_{reg}(\cdot )$ are realized by separate Cls and Reg convolutional branches together with a learnable scale factor to stabilize regression at different scales. During training, Focal Loss and CIoU Loss are used for classification and localization supervision.

With this design, the DSCD lightweight detection head preserves the simple three-scale prediction structure of YOLOv11n, while strengthening detailed feature modeling through 1×1 Conv_GN-based channel unification, a shared 3×3 Conv_GN tower and a decoupled Cls–Reg–scale head. This improves the detection of small and low-contrast rice lesions under complex paddy-field backgrounds, while keeping the number of head parameters and FLOPs suitable for real-time deployment on resource-constrained edge devices.

### 4.4. DepGraph Pruning

**Figure 5:**
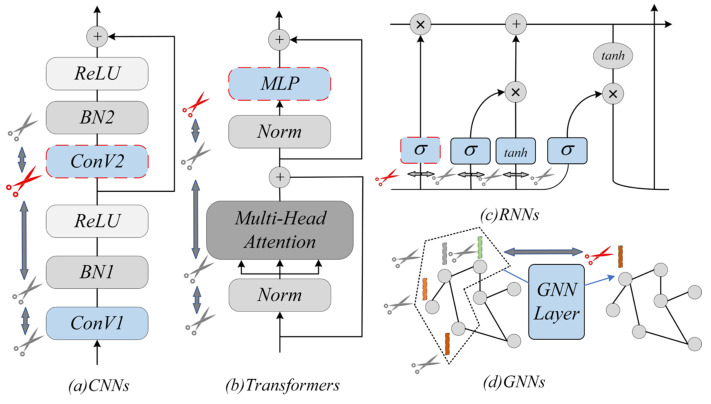
Illustration of parameter dependencies across layers in different network architectures, which require several layers to be pruned simultaneously.

When deploying SCD-YOLOv11n on edge devices, the numbers of parameters and FLOPs directly affect inference latency. Channel pruning is a common compression technique, but conventional methods often apply a single global pruning rate. Such uniform pruning can inadvertently remove channels that are crucial for small and low-contrast lesions, and may break residual or concatenation dependencies if the architecture is not handled carefully. To avoid these issues, we adopt DepGraph as a structured pruning framework and build a mixed group-normalization pruning rule on top of it. As illustrated in Figure 5, the DepGraph has demonstrated good generality and efficiency across various neural network architectures.

DepGraph represents convolution, normalization, residual and concatenation operators in a directed dependency graph. When a channel is pruned, the corresponding binary mask is propagated along the dependency edges so that all related kernels in the backbone, neck and head are pruned consistently. In this way, structural compatibility is preserved and the compressed model remains stable to train and deploy.

Within this framework, we use normalization statistics to define the importance of each channel. For the c-th channel, let $\gamma_{c}$ and $\sigma_{c}^{2}$ denote the scale parameter and running variance of its normalization layer, and define the importance score as:

(7) $$I_{c}=\left|\gamma_{c}\right|\sqrt{\sigma_{c}^{2}+\varepsilon }$$

where ε is a hyper-parameter that controls the mapping from normalized importance to the shrinkage factor. In our experiments, we set ε=4 so that the channel-wise shrinkage factor $\gamma_{c}$ lies approximately within the range [1, 16]. Channels in the same layer are sorted in descending order of $I_{c}$ and divided into K group.

(8) $$\;G_{1},G_{2},\ldots ,G_{K}$$

Each group $G_{k}$ is assigned a pruning ratio $\rho_{k}$: highly important groups use a smaller $\rho_{k}$, whereas less important groups use a larger $\rho_{k}$. Specifically, we remove the lowest-scoring $\rho_{k}$ fraction of channels within $G_{k}$ and rely on DepGraph to propagate the resulting masks and prune all dependent kernels along the graph. In practice, we divide the channels into three importance groups and set the pruning ratios to $\rho_{1}=0.1$, $\rho_{2}=0.3$ and $\rho_{3}=0.6$ for the high-, medium- and low-importance groups, respectively.

To control the overall compression level, we introduce a global speed-up factor defined as the desired ratio between the original and pruned models in terms of computational cost or parameter count. A larger speed-up factor corresponds to a more aggressive pruning configuration, leading to a higher effective pruning ratio and a more compact network.

In practice, we adopt a one-shot pruning procedure on the pretrained SCD-YOLOv11n baseline with a global speed-up factor of 2.0. Under this configuration, the parameter counts and FLOPs are reduced by roughly half, and the resulting model, which retains about 50% of the original channels, is used as the student network for subsequent channel-wise distillation.

### 4.5. Feature Distillation of CWD

After DepGraph pruning, SCD-YOLOv11n retains about 50% of the original channels and becomes suitable for edge deployment, but its reduced capacity may weaken the detection of small and low-contrast lesions. To compensate, we adopt a teacher–student scheme in which a YOLOv8n detector trained on the same rice disease dataset acts as the teacher and the 50% channel-pruned SCD-YOLOv11n serves as the student. The student is optimized under a joint detection–distillation objective.

For feature-level distillation, we employ Channel-wise Knowledge Distillation (CWD) on multi-scale neck features. Let $Y_{T}^{\left(l\right)},Y_{S}^{\left(l\right)}\in R^{C_{l}\times H_{l}\times W_{l}}$ be the feature maps at the l-th neck layer of the teacher and student, respectively. Because pruning changes the student’s channel width, we insert lightweight 1×1 convolutional alignment modules followed by non-affine normalization so that aligned student features match the teacher in channel dimension. For each aligned pair, CWD converts the c-th channel into a spatial probability distribution using a temperature-scaled softmax:

(9) $$\;\phi_{T}^{\left(c,l\right)}\left(i\right)=\frac{\exp\left(Y_{T,c,i}^{\left(l\right)}/T_{d}\right)}{\sum_{j=1}^{H_{l}W_{l}}exp\left(Y_{T,c,j}^{\left(l\right)}/T_{d}\right)},\phi_{S}^{\left(c,l\right)}\left(i\right)=\frac{\exp\left(Y_{S,c,i}^{\left(l\right)}/T_{d}\right)}{\sum_{j=1}^{H_{l}W_{l}}exp\left(Y_{S,c,j}^{\left(l\right)}/T_{d}\right)}\;$$

where i indexes spatial positions, $c=1,\ldots ,C_{l}$, and $T_{d}=1$ is the distillation temperature.

The CWD loss is defined as the asymmetric Kullback–Leibler divergence accumulated over all selected layers:

(10) $$L_{CWD}=T_{d}^{2}\sum_{l}\sum_{c=1}^{C_{l}}\sum_{i=1}^{H_{l}W_{l}}\phi_{T}^{\left(c,l\right)}\left(i\right)log\frac{\phi_{T}^{\left(c,l\right)}\left(i\right)}{\phi_{S}^{\left(c,l\right)}\left(i\right)}\;$$

Because each term is weighted by $\phi_{T}^{\left(c,l\right)}(i)$, positions where the teacher responds strongly are matched more strictly, while background locations contribute little. This encourages the pruned student to use its limited capacity to align with rice-lesion patterns rather than soil, water and weeds.

The student is trained with the standard detection loss $L_{det}$ plus the CWD loss:

(11) $$\;L_{total}=L_{det}+\lambda_{feat}L_{CWD}$$

where $\lambda_{feat}>0$ balances detection supervision and feature distillation. In our implementation, we set $\lambda_{feat}=1.5$ and keep this coefficient constant throughout training.

**Figure 6:**
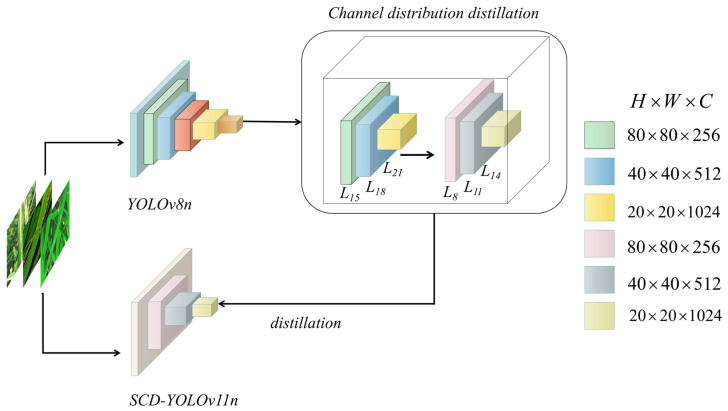
Schematic diagram of the channel-wise knowledge distillation process.

In practice, we select three multi-scale neck feature maps $L_{15},L_{18},L_{21}$ from the teacher, with spatial resolutions and channel widths 80×80×256, 40×40×512 and 20×20×1024, respectively. The student uses the corresponding layers $L_{11},L_{14},L_{8}$ as distillation targets, as illustrated in Figure 6. Restricting CWD to these three key scales transfers rich multi-scale information while keeping the additional cost of distillation moderate.

## 5. Results

### 5.1. Experimental Environment and Key Parameter Settings

The experimental environment and key parameters used in this study are summarized in Table 1.

This experimental investigation employs the following configuration: a batch size of 8 and input images resized to 640 × 640 pixels. To mitigate overfitting, a weight decay factor of 0.0005 is incorporated, and the Mosaic augmentation technique is implemented during the training pipeline to enhance the model’s generalization. The learning rate is managed by a cosine annealing scheduler, initialized at 0.01 and decaying to a terminal value of 0.01. The network is optimized using stochastic gradient descent (SGD) with a momentum parameter of 0.937, and the entire training procedure spans 300 epochs.

### 5.2. Evaluation Indicators

This study focuses on rice leaf disease detection in field scenes. Therefore, the evaluation protocol considers both detection accuracy and computational efficiency to reflect the suitability of the model for edge deployment in intelligent agriculture.

Model performance is assessed using several quantitative metrics. For detection accuracy, we adopt Precision (P), Recall (R), Average Precision (AP) and mean Average Precision (mAP). Precision measures the proportion of correctly identified diseased regions among all regions predicted as diseased, while Recall measures the proportion of actual diseased regions that are successfully detected. The formal definitions of these metrics, together with AP and mAP, are given in Equations (12)–(15).

(12) $$\;P=\frac{TP}{\left(TP+FP\right)}$$

(13) $$R=\frac{TP}{\left(TP+FN\right)}$$

(14) $$\;AP=\int_{0}^{1}P\left(R\right)dR\;$$

(15) $$\;mAP=\frac{1}{N}\sum_{i=1}^{N}AP_{i}\;$$

Key terms are defined as follows: True Positives (TP) correspond to correctly identified diseased regions; False Positives (FP) denote healthy regions erroneously flagged as diseased; and False Negatives (FN) indicate diseased regions that the model failed to detect. Average Precision (AP), calculated as the area under the Precision-Recall curve, measures the detection accuracy for an individual class. Consequently, the mAP metric is computed as the mean of AP values across all N disease categories.

For computational efficiency, we report the number of parameters, GFLOPs and model size to characterize computational and storage costs, and additionally report Frames Per Second (FPS) to measure recognition speed. These indicators jointly describe the trade-off between accuracy and latency for different models.

To quantify the generalization ability of the detector on external datasets, we further report the performance drop between our in-domain test set and an external rice disease dataset. Given the mean Average Precision values measured on our own test set $mAP_{in}$ and on an external dataset $mAP_{ext}$, the cross-dataset degradation is defined as:

(16) $$\Delta mAP=mAP_{in}-mAP_{ext}$$

where we report both ΔmAP@50 and ΔmAP@50:95 in percentage points.

### 5.3. Ablation Experiments

Ablation experiments on the rice disease dataset, summarized in Table 2, show how each module of SCD-YOLOv11n shapes the accuracy-latency trade-off. Replacing the original backbone with StarNet reduces parameters and FLOPs by roughly one quarter, with almost unchanged mAP@50 but about a two-point drop in mAP@50:95, indicating that backbone compression alone undermines precise localization of small, low contrast lesions. Adding the C3k2 Star neck largely recovers this loss, improving mAP@50:95 by more than one point at essentially the same computational cost, which strengthens multi-scale modeling of slender disease spots along rice leaves. Introducing the DSCD lightweight head brings a further, smaller increase in high IoU accuracy while cutting computation by around ten percent, which eases real-time field inference. On this basis, DepGraph pruning with the mixed group-normalization rule reduces parameters and FLOPs by about half and increases inference speed by roughly fifty percent, with only a marginal reduction in mAP@50:95. Finally, applying CWD feature distillation to the pruned model raises both mAP@50 and mAP@50:95 by about one point without adding computation, restoring performance to a level comparable to the uncompressed baseline. These stepwise changes show that the full SCD-YOLOv11n design yields an accuracy latency tradeoff that is better than that of the original YOLOv11n under the same hardware and input resolution on the rice disease dataset.

### 5.4. Comparative Experiments

#### 5.4.1. Comparative Analysis of Model Performance with Global Pruning Enabled and Disabled

The core of pruning lies in balancing detection accuracy and model efficiency. Global pruning uses a unified threshold to substantially compress parameters and computation, but may also remove key channels and thus affect accuracy. Non-global (local) pruning tends to preserve more important channels, leading to a smaller compression ratio but better retention of discriminative ability. Experimental results show that, with appropriate use of the proposed group-norm pruning rule, parameters and FLOPs can be significantly reduced while mAP remains essentially unchanged or is even slightly improved (Table 3).

#### 5.4.2. Effects of Different Pruning Rates

**Figure 7:**
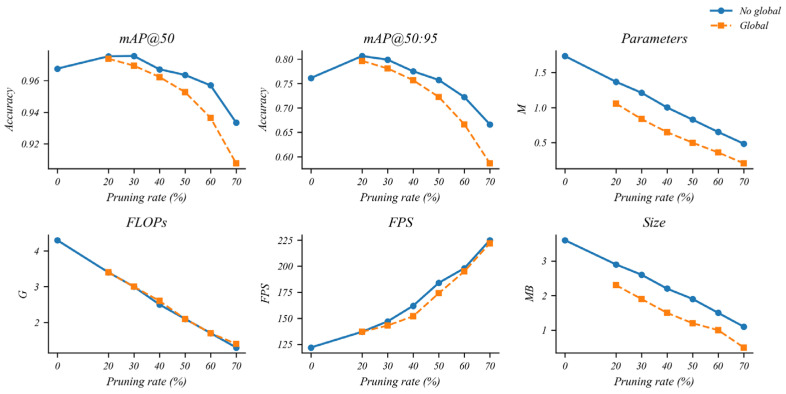
Performance of SCD-YOLOv11n under different pruning rates.

As illustrated in Figure 7, we further analyze the impact of different pruning rates by varying the target speed-up factor in the mixed group-normalization pruning rule. When the pruning rate is mild, the curves for parameter count and FLOPs drop noticeably, while the mAP curves change only slightly, indicating that a considerable amount of redundancy can be removed without clearly harming detection accuracy. As the pruning rate increases, the model becomes progressively more compact in all complexity indicators, but the mAP values also show a gradual decline, reflecting the inherent trade-off between efficiency and accuracy under aggressive compression. Overall, the trends in Figure 7 suggest that moderate pruning rates provide a balanced configuration for edge deployment—achieving substantial reductions in model size and computation while maintaining competitive detection performance—whereas very high pruning rates are more appropriate for scenarios with stricter resource constraints and tolerable accuracy loss.

Based on the above observations, we adopt the non-global pruning configuration with a 50% pruning rate as the student model for knowledge distillation. This setting reflects the main trade-off identified in the pruning analysis: it provides a clear reduction in parameters while retaining sufficient feature learning capacity for accurate detection.

#### 5.4.3. Comparison of Differences in the Number of Channels Across Layers Between Different Pruning Strategies

**Figure 8:**
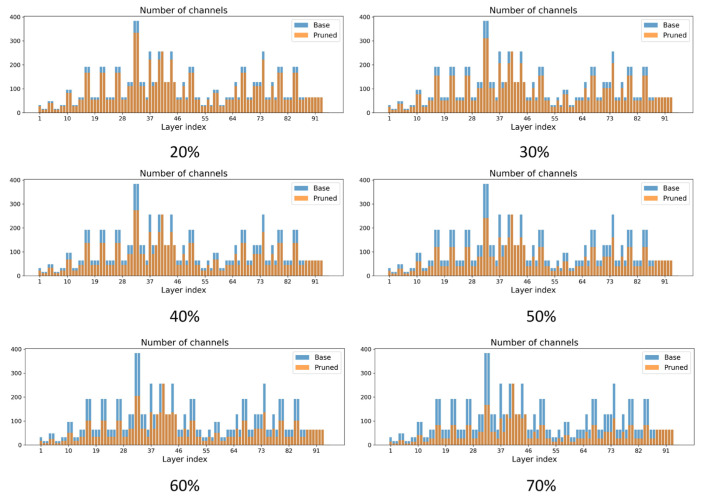
Channel distributions in locally pruned models under different pruning intensities.

Figure 8 compares the per-layer channel distributions of the base model and locally pruned models under different pruning strengths. With low pruning rates, the pruned networks remain close to the baseline, and channel reduction is mainly concentrated in intermediate layers. As the pruning rate increases, channels in middle and deeper layers are progressively thinned, while shallow feature extraction layers and the detection head retain most of their width. This is crucial for capturing fine-grained lesion textures and subtle color variations in complex paddy-field backgrounds. Under more aggressive pruning, many intermediate layers lose a large proportion of their channels, but shallow and output layers still remain relatively wide, indicating that the local pruning strategy produces a non-uniform, structure-aware sparsity pattern that preferentially compresses redundant interior channels while preserving key layers for reliable rice disease detection.

**Figure 9:**
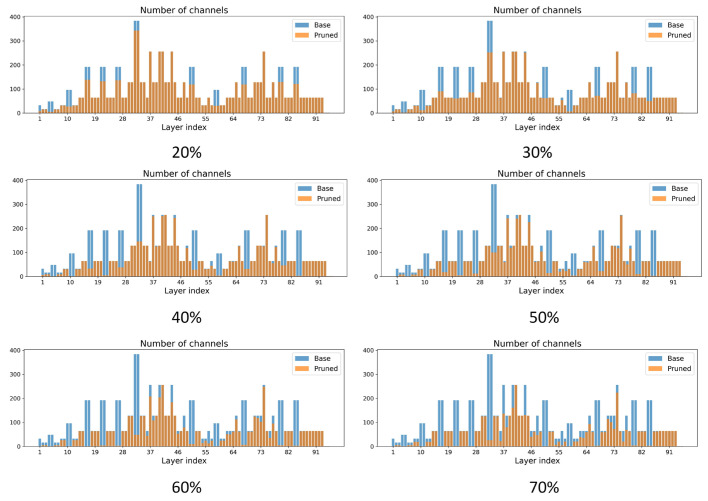
Channel distributions in globally pruned models under different pruning intensities.

Figure 9 summarizes the channel distributions of globally pruned models under different pruning strengths. The pruning pattern is clearly hierarchical and non-uniform: channels in shallow feature extraction layers change only slightly, so basic edge, texture and color cues in field images are largely preserved. In contrast, many mid- and late-stage refinement and fusion layers are strongly compressed, suggesting that these regions contain higher redundancy and therefore become the main targets of global pruning. Several attention blocks and bottleneck structures also exhibit noticeable channel reduction, indicating that the compression acts on the overall architecture rather than only on plain convolutional layers. Because channel retention is determined by a global importance distribution, deeper features can be reduced more aggressively to improve compression efficiency, while shallow and output layers retain sufficient width for stable lesion discrimination in practical rice disease detection.

#### 5.4.4. Comparative Analysis of Various Pruning Strategies

**Figure 10:**
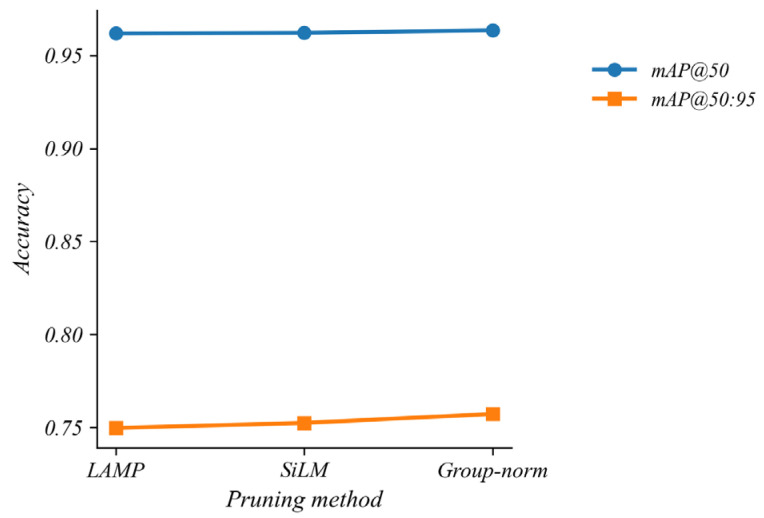
Performance of alternative pruning techniques.

To further assess the effectiveness of the proposed group-norm pruning technique, we compare it with the LAMP [26] and Slim [27] methods under the same 50% local pruning rate. As shown in Figure 10, the group-norm variant achieves higher detection accuracy than the two baselines at comparable compression levels, indicating that it provides a more favorable balance between compression and feature retention for rice disease detection in field images.

#### 5.4.5. Comparison of Different Feature Loss Weights

**Figure 11:**
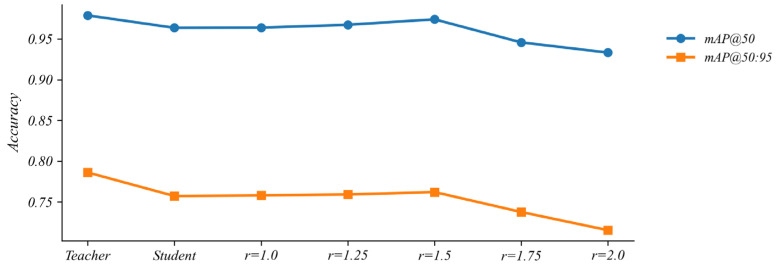
Effect of the feature-loss weight on model performance.

To investigate how the strength of feature distillation affects the performance of the compressed detector, we introduce a Feature Loss Ratio hyper-parameter to control the alignment strength between the features of the student and teacher models, while keeping all other training settings fixed to ensure a fair comparison. As shown in Figure 11, different values of this weight lead to changes in overall detection accuracy. Experimental results show that when the weight is set to 1.5, the model attains higher accuracy than the baseline pruned model, whereas substantially smaller or larger values are associated with lower accuracy.

#### 5.4.6. Comparison of Different Distillation Methods

**Figure 12:**
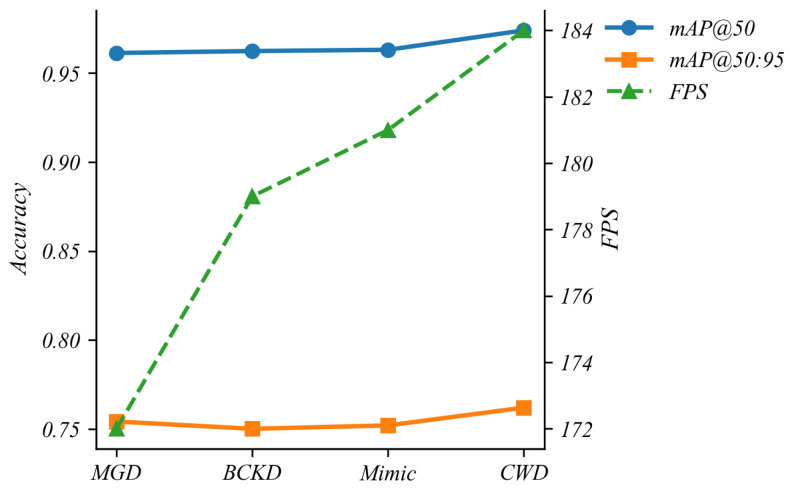
Performance of alternative distillation approaches.

To examine the effect of different distillation strategies on the pruned architecture, we compare four methods on the same 50%-channel SCD-YOLOv11n student with a YOLOv8n teacher, namely Matching-Guided Distillation (MGD) [28], Bidirectional Confidence Knowledge Distillation (BCKD) [29], Feature Mimicry (Mimic) [30] and Channel-Wise Distillation (CWD). All methods share identical pruning configurations, training schedules and data augmentation. For BCKD, the Logical Loss Ratio is set to 1.5, and for MGD and Mimic the distillation layers and related hyper-parameters follow their recommended settings, while the remaining configurations are kept consistent with CWD. As shown in Figure 12, under the same compression level CWD attains higher detection accuracy than the other methods, indicating that enforcing consistency on channel-wise feature distributions is more compatible with the structured channel pruning adopted in SCD-YOLOv11n. Therefore, CWD is used as the distillation strategy for the pruned student model.

#### 5.4.7. Comparative Evaluation of Detection Algorithms

In the comparative evaluation (Table 4), YOLOv5n exhibits the lowest detection accuracy, whereas YOLOv8n achieves the highest mAP values, improving mAP@50 and mAP@50:95 over YOLOv5n by about 0.31 while using roughly 13% more parameters and 5% more FLOPs. The intermediate baselines YOLOv10n, YOLOv11n and YOLOv12n increase mAP@50 by around 0.28 compared with YOLOv5n, while their parameter counts and FLOPs stay within about 25% of that model, so their runtime characteristics are of similar order.

To reflect design choices often used in rice and crop disease detection for small, low-contrast lesions, two YOLOv11n variants with BiFPN [31] and DySample [32] modules are further evaluated. Relative to YOLOv11n, the BiFPN variant reduces parameters by about one quarter and the DySample variant keeps parameters and FLOPs almost unchanged; in both cases, the changes in mAP and FPS are within a few percentage points. SCD-YOLOv11n raises mAP@50 by about 1.2 percentage points while keeping mAP@50:95 within 0.6 percentage points of YOLOv11n, and at the same time reduces parameters and FLOPs by roughly two thirds and increases FPS by more than 40%; it attains mAP@50 within about 0.5 percentage points and mAP@50:95 within about 2.5 percentage points of YOLOv8n while using over 70% fewer parameters and about three quarters fewer FLOPs.

In summary, under the edge device resource constraints defined in this study, SCD-YOLOv11n achieves a practical balance among detection accuracy, model size, computational cost and inference speed.

### 5.5. Visualization Analysis

**Figure 13:**
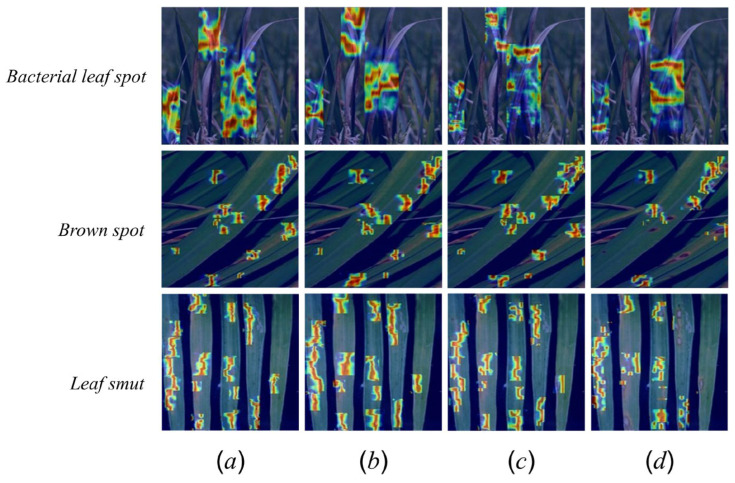
(**a**) SCD-YOLOv11n heat map; (**b**) YOLOv11n-DySample heat map; (**c**) YOLOv11n-BiFPN heat map; (**d**) YOLOv11n heat map. Warmer colors indicate higher activation.

To compare the spatial attention of different models on rice leaf diseases, we adopt the HiResCAM [33] method to generate high-resolution class activation heatmaps from the convolutional feature maps of the 10th, 12th, and 14th layers for SCD-YOLOv11n, YOLOv11n-DySample, YOLOv11n-BiFPN, and YOLOv11n. As shown in Figure 13, SCD-YOLOv11n produces high-response regions that are concentrated along diseased streaks or spots, with both lesion interiors and edges clearly highlighted; small and low-contrast lesions are still activated, while responses on healthy leaf areas and background structures remain weak. In the other variants, response patterns are more scattered, with blurrier lesion boundaries, occasional missed lesion areas, and noticeable activation on non-diseased regions.

This concentration of activations on lesion regions and suppression of background responses is consistent with the multi-scale feature extraction of the DSCD detection head and the structured pruning–distillation scheme, which together guide the model to focus more on disease-related patterns in the images.

### 5.6. Cross-Dataset Generalization on the RiceLeafDS Dataset

To assess the cross-dataset generalization ability of the proposed method on external data, we evaluate it on the Rice Leaf Disease Image Samples (RiceLeafDS) dataset, which serves as an external rice disease benchmark. For a fair comparison with our dataset, only the disease categories shared by both datasets, namely Bacterial leaf spot/Bacterial blight and Brown spot, are retained, while the remaining categories in RiceLeafDS are excluded from evaluation.

**Figure 14:**
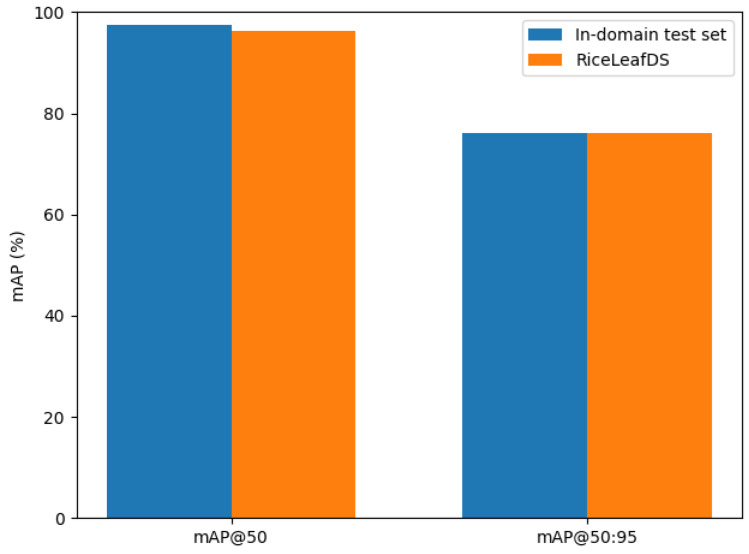
Performance comparison on the in-domain test set and the RiceLeafDS dataset.

The SCD-YOLOv11n model trained solely on a public rice disease dataset is directly applied to RiceLeafDS for inference, without any additional retraining or fine-tuning. The image preprocessing pipeline and input configurations are kept identical to those used in the in-domain experiments, and the cross-dataset performance change is measured with respect to the in-domain test set. As shown in Figure 14, mAP@50 decreases from 97.40% on our test set to 96.20% on RiceLeafDS, a drop of 1.20 percentage points. For mAP@50:95, the score decreases slightly from 76.21% to 76.15%, with a reduction of only 0.06 percentage points. Overall, the performance degradation on the external RiceLeafDS dataset is limited, indicating that the model can still achieve detection performance that is close to its in-domain results.

## 6. Discussion

SCD-YOLOv11n is proposed as an edge-oriented rice leaf disease detection framework in which the StarNet backbone, C3k2-Star neck, DSCD detection head and a DepGraph-guided mixed group-normalization pruning plus channel-wise distillation (CWD) strategy are jointly designed around lesion characteristics and deployment constraints. On the rice leaf disease dataset used in this study, SCD-YOLOv11n achieves high mAP while reducing parameters, FLOPs and inference time compared with YOLOv11n and several lightweight YOLO variants, demonstrating that the StarNet and C3k2-Star combination efficiently captures fine streak and spot patterns, and that DSCD together with the pruning–distillation schedule improves localization of small and low-contrast lesions while compressing the network in a lesion-focused rather than uniform manner.

Comparative experiments and cross-dataset evaluation on RiceLeafDS indicate that the proposed model provides a competitive accuracy–latency trade-off and retains most of its detection capability on an external rice disease dataset despite substantial compression. Current experiments are still restricted to three major rice leaf diseases and one main training dataset. Future work will enlarge the data scope, introduce more crop-specific detection baselines and further integrate SCD-YOLOv11n into smart agriculture workflows, including fixed monitoring stations, handheld diagnostic tools and UAV-based inspection systems.

## 7. Conclusions

In this study, we developed SCD-YOLOv11n, a lightweight rice leaf disease detection framework oriented toward edge deployment in smart agriculture. Experiments on a dedicated rice disease dataset show that the framework can maintain competitive detection accuracy while substantially reducing model complexity and inference delay, indicating that jointly considering task characteristics and resource constraints is a viable way to support real-time field monitoring. Given its compactness and throughput, the framework is suitable for integration into edge devices and existing disease management workflows. Future work will extend SCD-YOLOv11n to broader disease and environmental conditions and explore its use within multi-source monitoring and decision-support systems for sustainable rice production. 

## Figures and Tables

**Table 1:** Experimental setup and hardware specifications.

Name	Environmental Parameters
CPU	12th Gen Intel(R) Core (TM) i7-12700
GPU	NVIDIA T1000 (4 GB dedicated memory)
Memory	32 GB
Python version	3.10.16
Deep Learning Framework	PyTorch 2.2.2
CUDA	12.1
Mosaic probability	1
HSV hue range	0.015
HSV saturation range	0.7
HSV value range	0.4
Scale jitter	0.5
Box loss weight	7.5
Class loss weight	0.5
DFL loss weight	1.5
NMS IoU threshold	0.7

**Table 2:** Ablation study of the proposed modules in SCD-YOLOv11n.

	Baseline	A	B	C	D	E
StarNet		√	√	√	√	√
C3k2 Star			√	√	√	√
DSCD				√	√	√
group-norm					√	√
CWD						√
mAP@50	0.9623	0.9647	0.9680	0.9676	0.9636	0.9740
mAP@50:95	0.7682	0.7472	0.7584	0.7613	0.7572	0.7621
parameters	2,582,737	1,942,953	1,949,705	1,737,430	826,832	826,832
Flops/G	6.3	5.0	5.0	4.3	2.1	2.1
FPS	130	125	124	122	184	184
Size	5.2 MB	4.0 MB	4.0 MB	3.6 MB	1.9 MB	1.9 MB

**Table 3:** Comparison of local and global pruning strategies.

Pruning Method	Global-Prune	mAP@50	mAP@50:95	Parameters	Flops/G	FPS	Size
Base		0.9676	0.7613	1,737,430	4.3	122	3.6 MB
group-norm	False	0.9756	0.7988	1,212,092	3.0	147	2.6 MB
group-norm	True	0.9695	0.7809	835,998	3.0	143	1.9 MB

**Table 4:** Comparison of results of different algorithms.

Models	mAP@50	mAP@50:95	Parameters	Flops/G	FPS	Size
YOLOv5n	0.673	0.470	2,649,200	7.7	123	5.3 MB
YOLOv8n	0.9788	0.7861	3,006,233	8.1	137	6.2 MB
YOLOv10n	0.9613	0.7502	2,265,753	6.5	115	5.6 MB
YOLOv11n	0.9623	0.7682	2,582,737	6.3	130	5.2 MB
YOLOv12n	0.9587	0.735	2,508,929	5.8	115	5.2 MB
YOLOv11n-BiFPN	0.9642	0.7478	1,923,213	6.3	118	4.0 MB
YOLOv11n-DySample	0.9693	0.7515	2,595,089	6.3	125	5.3 MB
SCD-YOLOv11n(ours)	0.9740	0.7621	826,832	2.1	184	1.9 MB

## Data Availability

The rice disease detection dataset used in this study is publicly available at: https://universe.roboflow.com/dreamydaisy-cdagn/rice-dyl9n/dataset/4 (accessed on 10 December 2025). Additional original contributions presented in the study are included in the article, and further inquiries can be directed to the corresponding author.

## Supplementary Materials

The following supporting information can be downloaded at: https://www.mdpi.com/article/10.3390/s26010035/s1.

## Abbreviations

The following abbreviations are used in this manuscript:

mAP@50	AP at an IoU threshold of 0.50
mAP@50:95	mean AP over IoU thresholds from 0.50 to 0.95
CIoU	Complete Intersection over Union
DFL	Distribution Focal Loss
NMS	Non-Maximum Suppression
FLOPs	Floating-Point Operations
GFLOPs	Giga Floating-Point Operations
FPS	Frames Per Second
YOLO	You Only Look Once
SCD-YOLOv11n	proposed lightweight YOLOv11n-based rice disease detector with StarNet backbone, DSCD head, DepGraph pruning and CWD distillation
DSCD	Detail-Strengthened Cross-scale Detection head
DepGraph	Dependency-Graph-based structured pruning framework
CWD	Channel-Wise Distillation
LAMP	Layer-wise Adaptive Magnitude-based Pruning
MGD	Matching-Guided Distillation
BCKD	Bidirectional Confidence Knowledge Distillation
HiResCAM	High-Resolution Class Activation Mapping
Conv_GN	Convolution followed by Group Normalization
Cls	Classification branch of the detection head
Reg	Regression branch of the detection head
